# Patterns of PrEP continuation and coverage in the first year of use: a latent class analysis of a programmatic PrEP trial in Kenya

**DOI:** 10.1002/jia2.26137

**Published:** 2023-07-04

**Authors:** Kenneth K. Mugwanya, Adam Palayew, Torin Schaafsma, Elizabeth M. Irungu, Elizabeth Bukusi, Nelly Mugo, Jennifer Morton, Josephine Odoyo, Kenneth Ngure, Jared M. Baeten, for the Partners Scale‐Up Project

**Affiliations:** ^1^ Department of Epidemiology University of Washington Seattle Washington USA; ^2^ Department of Global Health University of Washington Seattle Washington USA; ^3^ Centre for Clinical Research Kenya Medical Research Institute Nairobi Kenya; ^4^ Department of Obstetrics and Gynecology University of Washington Seattle Washington USA; ^5^ Centre for Microbiology Research Kenya Medical Research Institute Nairobi Kenya; ^6^ Department of Community Health Jomo Kenyatta University of Agriculture and Technology Juja Kenya; ^7^ Department of Medicine University of Washington Seattle Washington USA; ^8^ Gilead Sciences Foster City California USA

**Keywords:** adherence, HIV prevention, Kenya, PrEP, retention, risk factors

## Abstract

**Introduction:**

Effective PrEP use is critical for impact, but data are limited on common patterns of continuation and coverage among persons using PrEP in real‐world settings.

**Methods:**

Data are from the Partners Scale‐Up Project, a programmatic stepped‐wedge cluster‐randomized trial to integrate PrEP delivery in 25 Kenyan public health facilities conducted between February 2017 and December 2021. We evaluated PrEP continuation using visit attendance and pharmacy refill records, and computed medication possession ratio to define coverage during the first year of use. Latent class mixture models were used to identify and characterize membership to different PrEP continuation patterns. Multinomial logistic regression was used to examine the association between group trajectories and demographic and behaviour characteristics.

**Results:**

Overall, 4898 persons initiated PrEP, 54% (2640) were female, mean age was 33 years (standard deviation 11) and 84% (4092) had partners living with HIV. PrEP continuation was 57%, 44%, and 34% at 1, 3, and 6 months, respectively. Four unique trajectories of PrEP coverage were identified: (1) one‐fourth (1154) exhibited consistent high coverage throughout the year with 93%, 94%, 96%, and 67% continuing PrEP at months 1, 3, 6, and 12, respectively; (2) 13% (682) showed high coverage trajectory throughout 6 months but coverage rapidly declined thereafter (94%, 93%, 63%, and 10% continued at months 1, 3, 6, and 12, respectively); (3) 18.9% (918) exhibited moderate coverage trajectory with 91% of clients refilling PrEP at month 1 but nearly all dropped‐off thereafter (37%, 5%, and 4% continued at months 3, 6, and 12, respectively); and (4) 43.8% (2144) exhibited immediate discontinuation trajectory, in which nearly all did not have any subsequent PrEP refill. Overall, being female, older age, having partners living with HIV or of unknown HIV status were statistically associated with better PrEP continuation trajectories compared to the immediate discontinuation trajectory (*p* <0.05 for all).

**Conclusions:**

In this analysis of a real‐world PrEP implementation programme in Kenya, we found four distinct patterns of PrEP continuation, with one‐third of users exhibiting consistent high continuation throughout 12 months and two‐fifths with immediate discontinuation patterns. These data may help guide tailored interventions to support PrEP continuation in this setting.

## 1 INTRODUCTION

Oral pre‐exposure prophylaxis is a highly effective HIV prevention strategy for men and women when taken with its effectiveness highly dependent on adherence [1, 2, 3]. Indeed, across first‐generation clinical PrEP trials, variations in HIV protection (0%–75%) in these studies highly correlated with the reported objective adherence level based on the quantification of drug concentrations in blood [1, 2, 3, 4, 5, 6]. Follow on PrEP implementation projects in East and Southern Africa have been characterized by high PrEP uptake followed by a steep drop‐off in persistence over time [7, 8, 9, 10, 11, 12, 13], especially among adolescent girls and young women. Some studies, however, have observed low HIV incidence despite reporting imperfect but reasonable adherence [7, 8, 10, 14, 15] highlighting the need to better understand how clients align prevention choices with risk —referred to as prevention‐effectiveness [16]—to better define the success of PrEP programmes.

Access to oral PrEP in real‐world settings is gradually scaling up in many African settings, including in Kenya which officially launched PrEP implementation in May 2017 [17, 18]. For HIV prevention programmes, effective PrEP use is not only important to reduce risk at an individual level, but it is essential to attain the overall population‐level impact of PrEP. Thus, there is a need to understand common patterns of PrEP continuation in real‐world settings in diverse at‐risk populations to better define programme success. The goal of this analysis was to assess patterns of PrEP continuation and medication possession as a measure of coverage and factors associated with those patterns in the first year of PrEP use among persons who initiated PrEP in a large real‐world implementation project delivering PrEP as part of the Kenya national PrEP programme [11, 19].

## 2 METHODS

### 2.1 Setting and population

Data are from the Partners Scale‐Up Project (ClinicalTrials.gov: NCT03052010) which has previously been reported [11, 19]. Briefly, the Partners Scale‐up project was a stepped‐wedge cluster‐randomized programmatic trial to catalyse scale‐up of PrEP delivery integrated in 25 high‐volume public HIV care clinics in Central and Western Kenya regions. The study was conducted between February 2017 and December 2021, as part of the Kenya National PrEP rollout programme [11]. The programme targeted at‐risk persons in serodifferent partnerships but any clinically eligible person seeking PrEP could access it [19]. The multi‐faceted strategies to promote the integration of PrEP at participating clinics included training of existing healthcare providers, provision of ongoing technical assistance to mentor and coach healthcare providers, and stakeholder engagement at the national and county level. After the training of healthcare providers, clinics started implementing PrEP delivery in a staged fashion.

### 2.2 Procedures for PrEP initiation

The primary objective of the project was to sustainably catalyse scale‐up of PrEP delivery and to study the process of integrating PrEP services within existing public health structures. Thus, all direct PrEP delivery services, including PrEP demand creation, HIV risk assessment, PrEP eligibility determination, HIV testing, counselling, prescribing, dispensing and client follow‐up activities were fully performed by existing Ministry of Health (MOH) healthcare providers, without additional financial support beyond training and technical assistance. Healthcare providers typically included clinical officers, nurses, HIV counsellors and pharmacy technicians already stationed at the implementing clinics. PrEP eligibility was determined according to the Kenya MOH PrEP guidelines [17] using the MOH PrEP Rapid Assessment Screening Tool which identifies the presence of any key behavioural factors in the last 6 months as an indication for substantial ongoing risk of acquiring HIV necessitating counselling for PrEP (File S1). PrEP was supplied from the Kenya Medical Supplies Authority and was provided for free to clients as part of standard of care.

### 2.3 Procedures for follow‐up of clients on PrEP

Clients who initiated PrEP were followed according to the Kenya national PrEP guidelines [17], which recommend follow‐up visits at 1 month and then quarterly after initiation. Although PrEP is prescribed for 3 months for clinical review, clients often complete monthly refill visits (i.e. without the need to see a clinician) in between to manage the PrEP medication stock and programme costs. Due to facility‐level contextual modifications [20], most facilities chose to dispense PrEP medication that exceeded the monthly supply recommended by the MOH guidance to help improve client convenience and continuation. Strategies to foster client continuation reflected those that are doable and successful in the Kenyan public health context and mostly included but were not limited to reminder phone calls.

### 2.4 Data collection

For this programmatic project, all clinical and programme data were documented by the facility staff on MOH tools that include PrEP registers and the clinical encounter form which is the standard MOH form for documenting individual‐level clinical records for clients on PrEP, and pharmacy logs for tracking drug prescriptions and dispensing. Over the study duration, PrEP client records, including demographics, HIV behaviour risk assessment and PrEP eligibility, PrEP prescription, dispensing, visit attendance and refills were abstracted off the standard MOH tools typically on a weekly basis by trained project‐dedicated staff into a central database using the SurveyCTO platform. For data quality assurance, we conducted several rounds of data cleaning exercise termed “data reconciliation” which involved project‐dedicated staff triangulating all MOH data sources to ascertain the correctness and completeness of data records. Given the programmatic nature of the data collection, the records reconciliation process focused on ensuring completeness of selected key variables that included: visit attendance, PrEP prescription and dispensing dates, number of PrEP pills/bottles dispensed, HIV test results, de‐identified unique client code, and all baseline demographics and HIV risk behaviours. Discrepancies in data on these records were resolved by confirmation from the facility healthcare providers.

The Partners Scale‐Up Project was reviewed and approved by the University of Washington Human Subjects Division, Seattle (STUDY00002183) and the Scientific and Ethics Review Unit of the Kenya Medical Research Institute (SERU/CCR/0048/3328, SERU/CMR/0040/3338) enabling abstraction and analysis of de‐identified and delinked programmatic data. Thus, individual consent was not required for programmatic procedures [11].

### 2.5 Analysis

The present analysis was restricted to the individuals’ first year of PrEP use. We conducted secondary data analysis of the parent Partners Scale‐Up Project to identify trajectories of PrEP continuation and coverage and factors associated with assignment to the different PrEP continuation trajectories. The primary outcomes were PrEP continuation and PrEP medication possession ratio (MPR) as a measure of coverage. Continuation was defined as documented visit attendance and or PrEP dispensing from pharmacy records. Refill visits completed in the period between 14 days before the expected visit date to 15 days before the next expected visit date were classified as attended. The MPR, referred to as coverage hereafter, was estimated for fixed 30‐day monthly (aligned with 30 pills per bottle), quarterly and annual periods to help account for cycling on and off PrEP. Coverage as measured by the medication possession was computed as the ratio of the total number of pills dispensed at last visit divided by the number of days in each computed period since PrEP dispensing (i.e. monthly, quarterly or annual). Coverage greater than 1 (i.e., indicating the client came back for a PrEP refill before the expected date with pills ran out) was truncated to 1, for each computed period. If a client did not have PrEP in possession, they were considered not covered during that period. For example, if a client was prescribed 30 PrEP tablets but returned for refill after 90 days (3 months), they would be considered fully covered in the first month but not covered in the second and third months and their 3‐month coverage during that period will be computed as 30 out of 90 days (or ∼33%). Dropout and non‐visit attendance were primary study outcomes and thus not treated as missing data. Although it is not possible to fully ascertain or test the true data‐generating process on the PrEP discontinuation outcome [21], we assumed missing at random mechanism to be quite a reasonable assumption [21] because our data reconciliation process resulted in <1% incompleteness on the key baseline and HIV risk behaviour variables for the 4898 persons to support full‐information maximum likelihood estimation.

We fit latent class mixture models (LCMMs) to identify distinct patterns of coverage in this study population [22]. LCMMs are a flexible family of models that are able to distinguish different longitudinal patterns from empirical data [23]. These models allow individuals to be assigned to groups based on their outcome over time in the context of how it compares to the longitudinal patterns in the data [24]. We fit several LCMMs to the data to identify the model with a clinically meaningful number of group trajectories accounting for the possibility of 1–4 classes. We examined model fit based on our practical and theoretical understanding of determinants of PrEP continuation in this population and the following statistical criteria: (1) the Bayesian information criterion (BIC), with lower BIC indicating better model fit; (2) Akaike information criterion (AIC), with lower AIC indicating better model fit; (3) likelihood, with higher log‐likelihood indicating better model fit; and (4) entropy, with values >0.8 suggesting a better distinction between classes. A model with four‐group classes was selected File (S2). After we identified the best class model, we then used the same criteria to compare linear, b‐spline, spline, quadratic spline and cubic spline forms for each trajectory. The best clinically meaningful model was a four‐group spline form model that was then used to assign each person membership to different patterns of continuation. We examined the distribution of demographic factors and behavioural risk characteristics in the different groups. Lastly, we used multinomial logistic regression models to examine the association between class membership and clinically relevant covariates determined apriori and included demographics, clinical and sexual behaviour variables, and HIV risk. Variables with a sample size <50 and those with >5% missingness—which included circumcision status for males and pregnancy or breastfeeding status for women—were not included in the model estimation. HIV risk was summarized using a composite empiric risk score developed among HIV serodiscordant couples [25]; a risk score of ≥3 was associated with an annual HIV incidence of >3% in previous studies. Data analyses were conducted in R using the Tidyverse, LCMM and nnet packages [23, 26, 27].

## 3 RESULTS

Overall, a total of 4898 individuals initiated PrEP in the Partners Scale‐Up project between February 2017 and June 2020; 2640 (54%) were female, mean age was 33 years (standard deviation 11), the majority were married and monogamous (75%), and 4092 (84%) reported to have a partner known to live with HIV (Table 1). Among those with partners living with HIV, 2845 (70%) had a composite HIV risk score of ≥3. A small proportion (≤1%) of the study population reported injection drug use, transactional sex or intimate partner violence.

**Table 1:** Baseline demographic and behaviour characteristics by group trajectory

	OVERALL	Consistent high continuation	High continuation	Moderate continuation	Immediate discontinuation
	(*N* = 4898)	(*n* = 1154)	(*n* = 682)	(*n* = 918)	(*n* = 2144)
	*n* (%)	*n* (%)	*n* (%)	*n* (%)	*n* (%)
Age—years, mean (SD)	33 [10]	36 [11]	34 [10]	33 [10]	32 [10]
Age categories					
*≤24*	969 (19.9)	148 (12.8)	121 (17.7)	187 (20.4)	513 (23.9)
*25–30*	1344 (27.4)	269 (23.3)	188 (27.6)	276 (30.1)	611 (28.5)
*30–35*	967 (19.7)	237 (20.5)	147 (21.6)	180 (19.6)	403 (18.8)
*>35*	1618 (33.0)	500 (43.3)	226 (33.1)	275 (30.0)	617 (28.8)
Gender					
*Female*	2640 (53.9)	671 (58.1)	369 (54.1)	483 (52.6)	1117 (52.1)
*Male*	2257 (46.1)	483 (41.9)	313 (45.9)	434 (47.3)	1027 (47.9)
*Transgender*	1 (0.0)	0 (0.0)	0 (0.0)	1 (0.1)	0 (0.0)
Marital status					
*Cohabiting*	219 (4.5)	36 (3.1)	14 (2.1)	39 (4.2)	130 (6.1)
*Married monogamous*	3678 (75.1)	917 (79.5)	552 (80.9)	704 (76.7)	1505 (70.2)
*Married polygamous*	569 (11.6)	161 (14.0)	75 (11.0)	101 (11.0)	232 (10.8)
*Never married*	347 (7.1)	24 (2.1)	32 (4.7)	56 (6.1)	235 (11.0)
*Separated or divorced*	68 (1.4)	15 (1.3)	9 (1.3)	12 (1.3)	32 (1.5)
*Widowed*	17 (0.3)	1 (0.1)	0 (0.0)	6 (0.7)	10 (0.5)
Circumcised (males only)	1584/1970 (80.4)	341/436 (78.2)	225/281 (80.1)	304/374 (81.3)	714/879 (81.2)
Number of children					
*No children*	888/2502 (35.5)	286/770 (37.1)	127/379 (33.5)	170/470 (36.2)	305/883 (34.5)
*1–2 children*	957/2502 (38.2)	254/770 (33.0)	152/379 (40.1)	194/470 (41.3)	357/883 (40.4)
*3+ children*	657/2502 (26.3)	230/770 (29.9)	100/379 (26.4)	106/470 (22.6)	221/883 (25.0)
Pregnant (females only)	167/1486 (11.2)	39/356 (11.0)	27/220 (12.3)	35/280 (12.5)	66/630 (10.5)
Breastfeeding (females only)	262/1687 (15.5)	67/435 (15.4)	28/246 (11.4)	55/313 (17.6)	112/693 (16.2)
HIV behaviour risk characteristics—last 6 months					
*Inconsistent condom use*	2817 (57.5)	724 (62.7)	361 (52.9)	507 (55.2)	1225 (57.1)
*Has sex partner living with HIV*	4092 (83.5)	1074 (93.1)	606 (88.9)	777 (84.6)	1635 (76.3)
*Sex partner at high risk of HIV or HIV status unknown*	789 (16.1)	98 (8.5)	90 (13.2)	136 (14.8)	465 (21.7)
*More than one sex partner*	565 (11.5)	78 (6.8)	60 (8.8)	98 (10.7)	329 (15.3)
*Intimate partner violence*	35 (0.7)	5 (0.4)	2 (0.3)	9 (1.0)	19 (0.9)
*Transactional sex*	67 (1.4)	3 (0.3)	7 (1.0)	13 (1.4)	44 (2.1)
*Recent STI*	44 (0.9)	7 (0.6)	2 (0.3)	11 (1.2)	24 (1.1)
*Recurrent use of post‐exposure prophylaxis*	55 (1.1)	18 (1.6)	5 (0.7)	6 (0.7)	26 (1.2)
*Sex under the influence of alcohol or drugs*	111 (2.3)	20 (1.7)	10 (1.5)	21 (2.3)	60 (2.8)
*Reported injection drug use*	5 (0.1)	2 (0.2)	1 (0.1)	2 (0.2)	0 (0.0)

### 3.1 Baseline demographic and behaviour characteristics by trajectory

Overall, among the 4898 persons who initiated PrEP, we identified four unique patterns of PrEP coverage during the first year of PrEP use classified as: (1) consistent high coverage, (2) high coverage, (3) moderate coverage, and (4) immediate discontinuation. The consistent high coverage group comprised 23.6% (1154) of the sample and demonstrated high levels of coverage throughout the 12‐month period. Clients in this group trajectory attended a median of 5 (IQR 4–7) follow‐up visits. The high coverage trajectory comprised 13.9% (682) of the sample and exhibited high coverage that persisted through the first month 6 followed by a rapid decline thereafter. Clients in this trajectory attended a median of 3 (IQR 2–4) follow‐up visits. The moderate coverage trajectory comprised 18.7% (918) of the sample and showed high coverage for only month 3 with coverage rapidly declining thereafter. Clients in this moderate trajectory attended a median of 2 (IQR 1–2) follow‐up visits. The immediate discontinuation group comprised 43.7% (2144) of the sample and exhibited immediate drop‐off in PrEP coverage with 0 (IQR 0–0) median number of visits attended after initiation, indicating that these were clients who initiated PrEP but never came back for a refill.

The general characteristics of clients assigned to each group trajectory using the maximum probability assignment rule are presented in Table 1. The majority (57%) of persons were assigned to three groups which continued PrEP for at least 3 months (i.e. the consistent high coverage, high coverage, and moderate coverage groups). Clients assigned to the consistent high coverage or high coverage trajectories were frequently older >45 years, while clients ≤24 years were mostly assigned to the immediate discontinuation group. A higher proportion of females were assigned to either the consistent high (58%) or high coverage (54%) trajectories than to the moderate coverage (52%) or immediate discontinuation trajectories (52%). Notably, a higher proportion of individuals with partners known to live with HIV were mostly in the consistent high PrEP coverage trajectory (93%), high coverage group (85%), and moderate coverage trajectories (85%) compared to the immediate discontinuation (76%). We also found that reported inconsistent condom use in the previous 6 months occurred at a similar frequency in the two major groups (i.e. 58% in the consistent high continuation group vs. 57% in the immediate discontinuation group), but sex with more than one partner or having sex with a partner with factors associated with high HIV risk or of unknown HIV status was more prevalent among persons in the immediate discontinuation trajectory compared to the consistent high continuation trajectory (Table 1).

### 3.2 Patterns of coverage and continuation on PrEP by identified group trajectory

**Figure 1:**
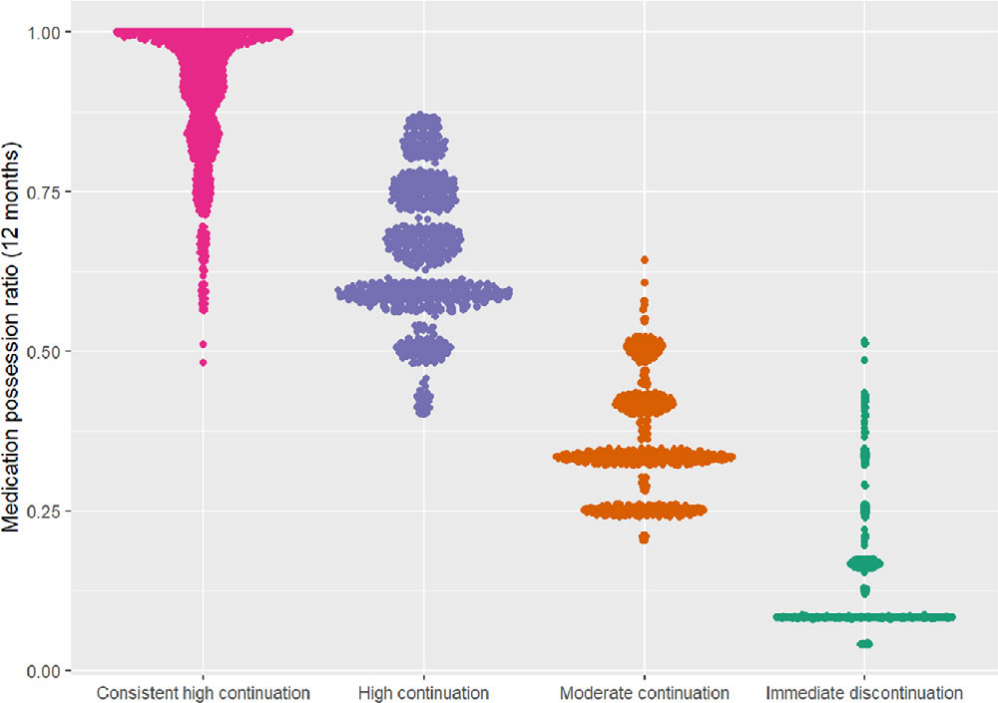
Violin scatter plot of coverage (MPR) over 12 months, by group trajectory. *Presents the distribution of the PrEP coverage over the 12‐month period since initiation for each individual in the sample by class trajectory.

**Figure 2:**
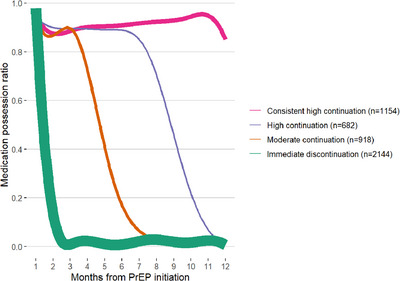
Profile of PrEP coverage from the latent class mixture model.

The graphic and quantitative representation of the four distinct trajectories of PrEP coverage are shown in Table 2, Figure 1, and Figure 2. Overall, among 4898 persons who initiated PrEP, 57%, 44%, 34%, and 23% returned for PrEP refill visits at 1, 3, 6, and 12 months post PrEP initiation, respectively (Table 2). At all quarterly visits, about 11%–14% of clients who had failed to attend the prior refill visit returned for PrEP medication. For the entire study population, the overall coverage in the first year of use was 41.7%, but there was considerable variation by trajectory (Figure 1): 91.3% for the consistent high coverage group, 64.9% for high coverage trajectory, 34.8% for moderate coverage and 10.6% for the immediate discontinuation trajectory. The patterns and shape of the smoothed curves for the four trajectories are presented in Figure 2. The consistent high coverage trajectory exhibited high mean quarterly PrEP coverage that remained consistently above 90% for most part of the first 12 months of use. This coverage pattern was consistent with the exceptionally high proportion of clients attending PrEP refill visits in that group: 93% at month 1, 95% at month 3, and 67% at month 12, respectively (Table 2).

**Table 2:** Quantitative summary of quarterly PrEP coverage and continuation by group trajectory

Outcome	Overall (*n* = 4898)	Consistent high (*n* = 1154)	High (*n* = 682)	Moderate (*n* = 918)	Immediate discontinuation (*n* = 2144)
Drug coverage or MPR (Quarters)					
*1–3*	67.5%	90.6%	92.5%	90.7%	37.4%
*4–6*	42.2%	90.2%	88.2%	43.4%	1.1%
*7–9*	32.8%	92.1%	68.3%	3.2%	2.1%
*10–12*	24.5%	92.4%	11.5%	1.8%	1.9%
Continuation (Months)					
*1*	2806 (57%)	1071 (93%)	641 (94%)	839 (91%)	255 (12%)
*3*	2135 (44%)	1095 (95%)	633 (93%)	341 (37%)	66 (3%)
*6*	1661 (34%)	1111 (96%)	430 (63%)	43 (5%)	77 (4%)
*9*	1167 (24%)	1018 (88%)	65 (10%)	31 (3%)	53 (2%)
*12*	916 (19%)	769 (67%)	67 (10%)	368 (4%)	44 (2%)

Individuals in the high coverage trajectory also exhibited high mean quarterly PrEP coverage that was above 90% in the first 6 months of PrEP use but rapidly declined thereafter (Table 2); >90% attended months 1 and 3, and 63% at 6 months, respectively. The moderate coverage trajectory had a curve shape somewhat similar to that of the high coverage trajectory group, but the drop‐off happened earlier at 3 months in this group; 91% of the clients attended month 1 but this was followed with a sharp drop‐off in continuation and coverage by month 3. The immediate discontinuation trajectory was characterized by consistently very low PrEP coverage and continuation throughout the 12 months; only 12% of clients completed month 1. Importantly, beyond the smoothed overall curves, there was notable heterogeneity in individual client trajectories within the four groups (Figure S1).

### 3.3 Correlates of assignment to the respective trajectory class membership

**Figure 3:**
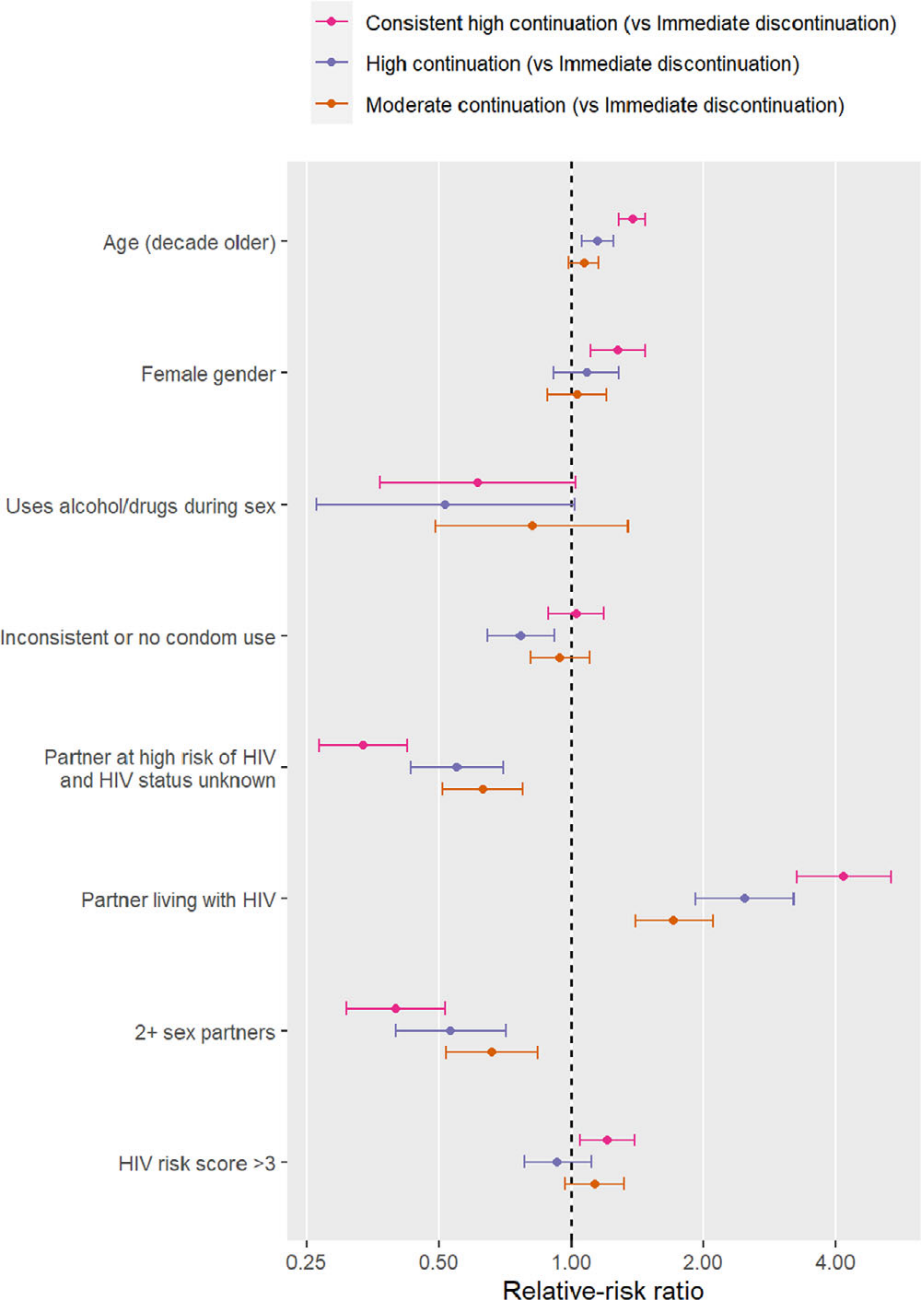
Forest plot of relative risk ratios and 95% confidence intervals of being assigned to each trajectory relative to the “immediate discontinuation” group trajectory for various baseline demographic and behaviour characteristics.

Results from the multinomial logistic regression model are presented in Figure 3. Overall, individuals of older age were significantly more likely to follow consistent high (RRR: 1.38, 95% 1.29–1.47) or high coverage (RRR: 1.15, 95% 1.06–1.25) trajectories compared to the immediate discontinuation trajectory. Females (RRR: 1.28, 95% 1.11–1.47) and those with a composite HIV risk score ≥3 (RRR: 1.21, 95% 1.05–1.39) were also more likely to belong to the consistent high coverage trajectory compared to the immediate discontinuation trajectory. Similarly, individuals with sexual partners living with HIV were significantly more likely to exhibit consistent high coverage (RRR: 4.18, 95% 3.26–5.35), high coverage (RRR: 2.48, 95% 1.92–3.21), and moderate coverage (RRR: 1.72, 95% 1.40–2.11) trajectories compared to the immediate discontinuation trajectory. In contrast, those who reported to have partners of unknown HIV status or those with multiple sex partners or reported sex under the influence of alcohol or drugs were significantly more likely to belong to the immediate discontinuation trajectory than in the consistent high, high, or moderate coverage trajectories (Figure 3).

## 4 DISCUSSION

In this evaluation of a large programmatic PrEP project in Kenyan public health HIV care clinics, we found moderate continuation on PrEP with a high drop‐off for most clients within 6 months post‐initiation that was characterized by four unique PrEP continuation patterns. More than a third of the individuals exhibited high continuation and coverage patterns on PrEP throughout the first year of use and about two fifth never returned for PrEP refill after initiation.

For HIV prevention as opposed to treatment, effective PrEP use is not lifelong but means aligning PrEP use with periods of elevated HIV risk objectively or perceived (i.e. prevention‐effectiveness adherence) [16], implying that individuals may cycle on and off as their vulnerability to HIV evolves. Thus, understanding reasons why users discontinue or return to PrEP use is not only necessary to define the effective use of PrEP at the individual level but is needed to appropriately define the success of PrEP programmes. We found that individuals having partners living with HIV and those with sexual behaviours likely to put them at elevated risk for HIV were more likely to exhibit high continuation and persistence patterns on PrEP. Consistent with previous studies [28, 29, 30, 31], these findings indicate that individuals continue reasonably well on PrEP when they perceive to be at risk for HIV—like having a partner living with HIV. Thus, efforts to identify strategies that support the recognition of HIV risk and normalization of PrEP use are needed to help persons navigate their decisions about PrEP use and HIV prevention preferences.

We found that differentiation of coverage and continuation patterns between the four trajectories emerged within 1 month post‐PrEP initiation consistent with patterns of persistence observed for daily medication in other prevention fields [32]. These data might imply that individuals try out new products and the majority make early decisions about whether to continue or discontinue possibly based on or modified by their perceived risk for HIV, safety concerns and opportunity costs, including client‐centeredness at service delivery points. The moderate coverage trajectory involved individuals who returned for at least one refill visit but did not return thereafter. Clients in this group present an opportunity to intervene, including interrogation of reasons for discontinuation to help guide targeted support strategies.

We previously demonstrated that for HIV serodifferent couples [33], using PrEP as a bridge until the partner living with HIV has been on antiretroviral therapy long enough to achieve viral suppression resulted in the elimination of HIV transmission within a partnership. In this study, most clients were in known serodifferent partnerships [11] and the observed patterns may reflect risk‐based decisions to stop PrEP due to change in risk either because of relationship resolution or the partner living with HIV had achieved viral load suppression. Specifically, we found that individuals in the high and moderate coverage trajectories exhibited patterns of continuation consistent with the implementation of PrEP as a bridge which would imply appropriate discontinuation of PrEP [30, 34]. Consistent with previous studies [7, 14], we also observed that individuals of younger age, those who are male or in unstable marital status were more likely to exhibit immediate discontinuation patterns on PrEP. Uptake and continuation on PrEP also depends on how well opportunity costs and health system barriers can be navigated by potential PrEP clients. In our previous work [31], we reported high enthusiasm for PrEP uptake but also found that major health system inefficiencies, including lengthy visits, endanger client continuation on PrEP. Additional effort is needed to document and understand the reasons for PrEP discontinuation to guide an appropriate definition of the success of PrEP programmes.

This work has several limitations. First, latent class analysis is an exploratory data‐driven evaluation for which, the emergent number of classes depends on the sample size and study time. In this analysis, the large study population permitted more than sufficient power to identify clinically meaningful trajectories. Second, the final class selection and naming of trajectories is based on the researcher judgement which may not be replicable [35]. Third, our analysis utilized programme data abstracted from client records which meant that only variables on the programmatic tools could be analysed. This limited the extent to which we could assess other important variables like clinic‐level factors, provider attitudes, stigma, sexual behaviour pattern and partner type, or perceived HIV risk that could impact a person's pattern of use [36]. Fourth, the sample population is only a representative of individuals in serodifferent relationship accessing PrEP in HIV clinics and may not be generalizable to other populations. PrEP was prescribed for daily dosing per the Kenya and WHO guidelines at the time and the MPR, as a measure of PrEP coverage, is only applicable to daily dosing schedule but would not be an appropriate metric if some individuals used non‐daily dosing schedules. Fifth, it is possible the observed coverage patterns may reflect appropriate discontinuation due to low‐risk situations, but this could not be ascertained without interviewing persons who discontinue. Lastly, continuation and the MPR represent refill adherence and pill possession, respectively, but not the ingestion of the quantity dispensed. Despite these limitations, our work provides important new data that can be used to guide targeted interventions to support users navigate modifiable barriers to access and use of PrEP for prevention in this setting. Importantly, it will be useful to understand whether similar analyses reveal comparable continuation patterns in different populations and settings in Kenya and elsewhere in African settings.

## 5 CONCLUSIONS

In this analysis from a large real‐world PrEP implementation programme in Kenyan facilities, we found evidence of distinct patterns of persistence and continuation on PrEP, with more than one‐third of the study population exhibiting patterns of consistent high PrEP continuation throughout the first 12 months of use. These findings may be useful in tailoring targeted interventions to support continuation and effective PrEP use in this setting.

## COMPETING INTERESTS

JMB is an employee of Gilead Sciences. KKM has received an Investigator Sponsored Research Grant from Gilead Sciences not related to this work awarded to the University of Washington. All other authors declare no competing interests.

## FUNDING

The study was supported by the National Institute of Mental Health of the US National Institutes of Health (grants R01MH095507, R00MH118134, R01MH123267 and R01AI155086) and the Bill & Melinda Gates Foundation (grant OPP10556051). The project funders had no role in the study design or writing of the report.

## AUTHORS’ CONTRIBUTIONS

Study conceptualization and funding acquisition: KKM and JMB. Data collection tool development: KKM, EMI, JM, and SP. Data collection: KKM, EMI, BN, KN, EB, NM, JO, JM, and SP. Project administration: KKM, EMI, JMB, EB, NM, JO, and JM. Data analysis: TS and AP. Writing—original draft: AP and KKM. Writing—review and editing: All authors. All authors participated in the critical review and have read and approved the final manuscript.

## Supporting information

Supplemental file 1: PrEP Rapid Assessment Screening Tool.

Supplemental file 2: Goodness of fit values for the different number of groups in the model.

Figure S1

## Data Availability

Public sharing of individual participant data was not included in the informed consent form of the project and cannot be posted in a supplemental file or a public repository because of legal and ethical restrictions. De‐identified data underlying this project can be made available to interested researchers upon reasonable request by contacting the International Clinical Research Center at the University of Washington (icrc@uw.edu).
