# Supplementary figures and images for: Patterns of PrEP continuation and coverage in the first year of use: a latent class analysis of a programmatic PrEP trial in Kenya
Source: J Int AIDS Soc. 2023 Jul 4;26(7):e26137. doi: 10.1002/jia2.26137 (PMC10320042; doi:10.1002/jia2.26137)

Supplemental file1: PrEP Rapid Assessment Screening Tool.


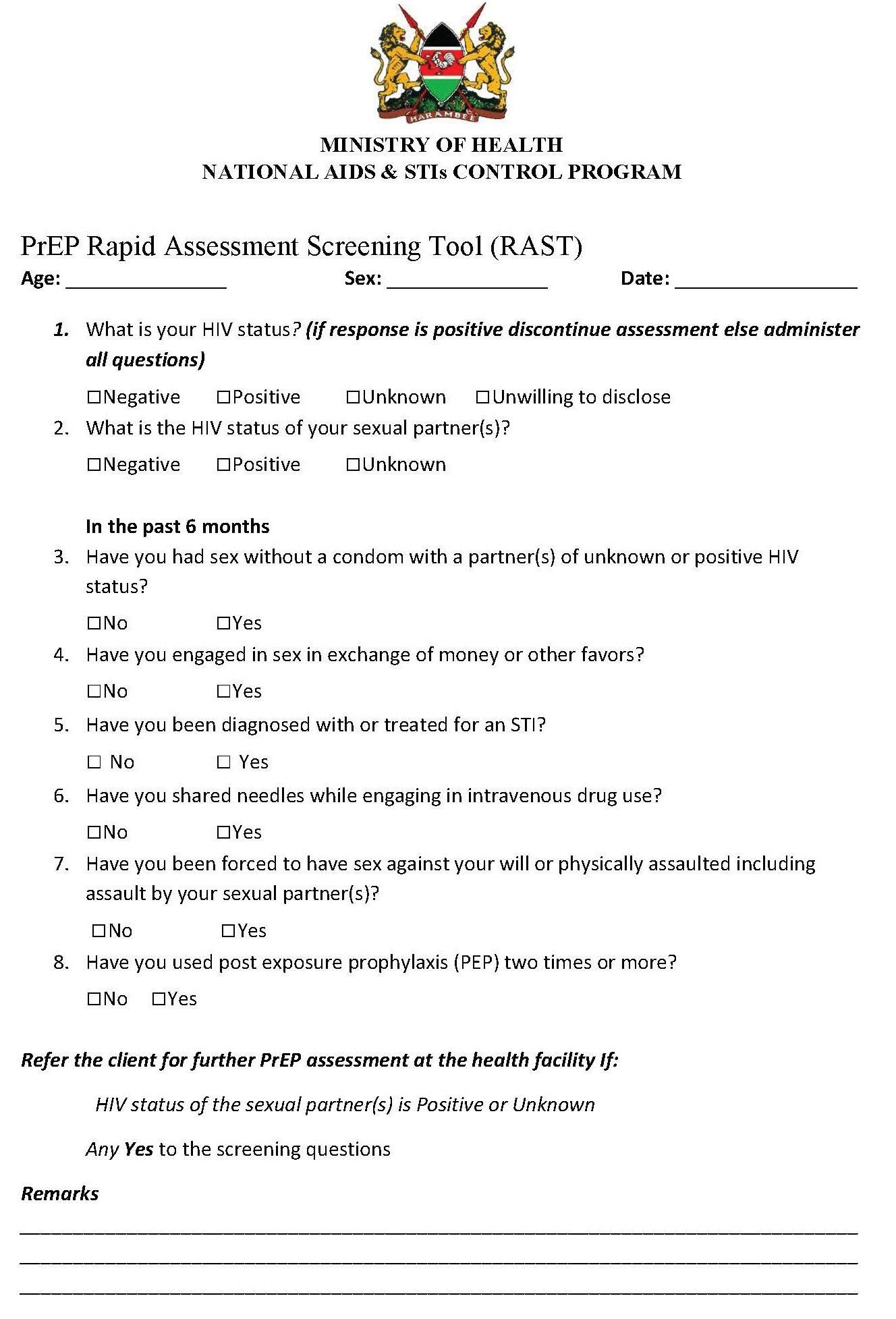

Supplement: Supplementary file 1 — Supplemental file 1: PrEP Rapid Assessment Screening Tool. [file JIA2-26-e26137-s002.docx]

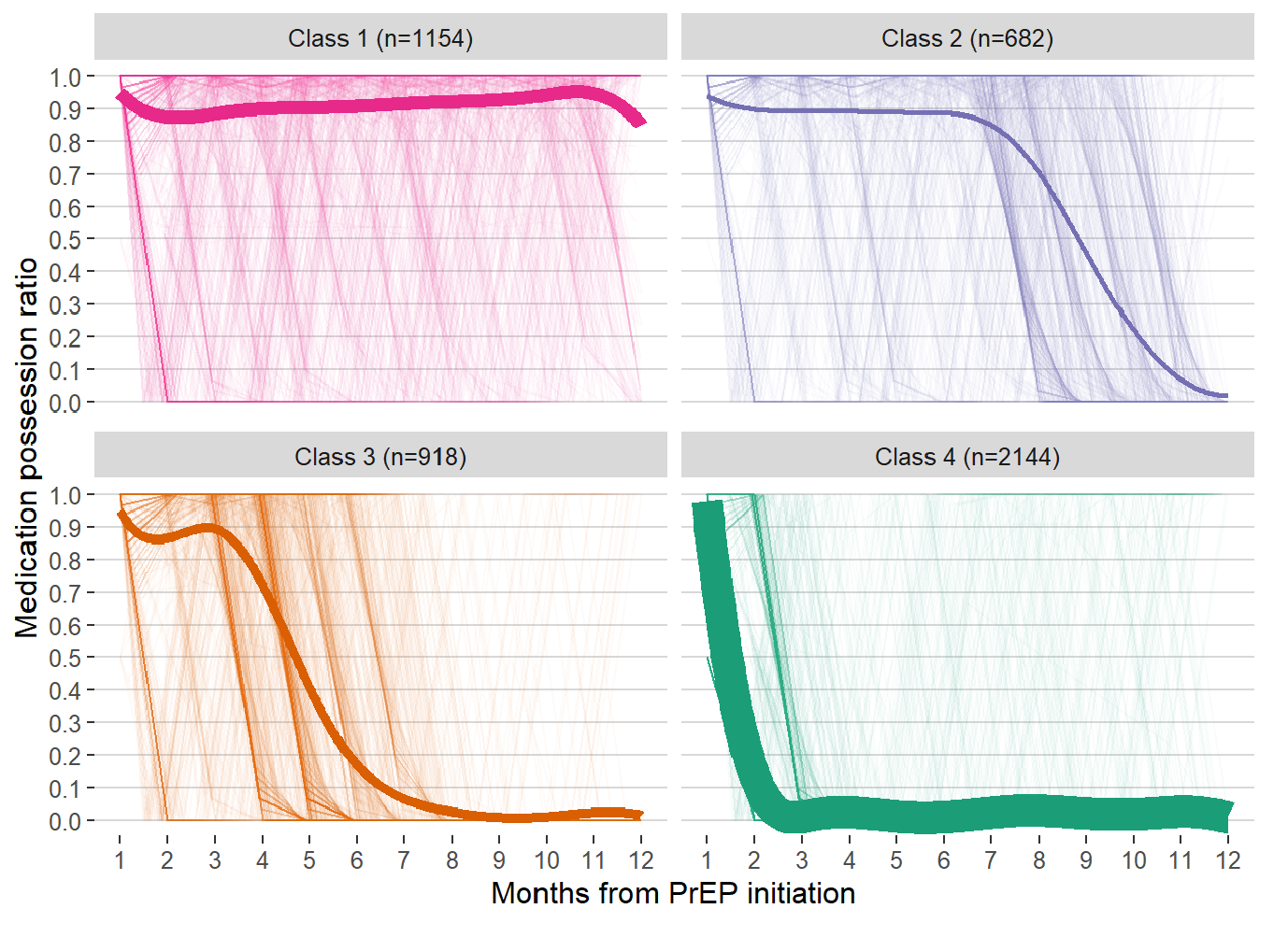

Supplement: Supplementary file 3 — Figure S1 [file JIA2-26-e26137-s001.tif]
