# Supplementary material for: Patterns of PrEP continuation and coverage in the first year of use: a latent class analysis of a programmatic PrEP trial in Kenya
Source: J Int AIDS Soc. 2023 Jul 4;26(7):e26137. doi: 10.1002/jia2.26137 (PMC10320042; doi:10.1002/jia2.26137)
Supplement: Supplementary file 2 — Supplemental file 2: Goodness of fit values for the different number of groups in the model. [file JIA2-26-e26137-s003.docx]

Supplemental file 2: Goodness of fit values for the different number of groups in the model.

| Model | Log Likelihood | AIC | BIC |
| --- | --- | --- | --- |
| 1 group | -6139.7 | 12309.4 | 12406.8 |
| 2 groups | 8137.6 | -16233.1 | -16096.7 |
| 3 groups | 8164.3 | -16274.6 | -16099.2 |
| 4 group | 18581.642 | -37097.28 | -36882.90 |
